# Burden and attributable risk factors of ischemic stroke in China from 1990 to 2019: an analysis from the Global Burden of Disease Study 2019

**DOI:** 10.3389/fneur.2023.1216777

**Published:** 2023-07-26

**Authors:** Yang Ye, Yu-Tian Zhu, Jia-Cheng Zhang, Hao-Lin Zhang, Rui-Wen Fan, Yu-Xin Jin, Hang-Qi Hu, Xi-Yan Xin, Dong Li

**Affiliations:** Department of Traditional Chinese Medicine, Peking University Third Hospital, Beijing, China

**Keywords:** burden, China, deaths, ischemic stroke, risk factor

## Abstract

**Background:**

The epidemiologic characteristics and attributable risk factors of ischemic stroke in China have changed over the past three decades. An up-to-date analysis on deaths, disability-adjusted life-years (DALYs), prevalence, incidence, and attributable risk factors of ischemic stroke for China is needed. This study aims to provide a comprehensive analysis of burden and attributable risk factors of ischemic stroke at national level in China by sex from 1990 to 2019.

**Methods:**

This is a secondary analysis of the Global Burden of Disease (GBD) study 2019. All data used in this study was derived from the 2019 GBD study. Deaths, DALYs, prevalence, incidence, and attributable risk factors of ischemic stroke in China by sex from 1990 to 2019 were analyzed.

**Results:**

From 1990 to 2019, the age-standardized deaths rate decreased by 3.3%, age-standardized DALYs rate decreased by 4%, age-standardized prevalence rate increased by 33.5%, and age-standardized incidence rate of ischemic stroke in China increased by 34.7%. In 2019, ambient particulate matter pollution became an important risk factor, whereas household air pollution from solid fuels was no longer a major risk factor for ischemic stroke in China. Burden of ischemic stroke was higher in China compared to other regions. Ambient particulate matter pollution among men, and diet high in sodium, smoking, household air pollution from solid fuels among women account for the increased deaths/DALYs due to ischemic stroke in China.

**Conclusion:**

Our study revealed that great changes have occurred in burden and attributable risk factors of ischemic stroke in China in the past three decades. Distinct sex-specific differences are observed in burden and attributable risk factors.

## 1. Introduction

Globally, stroke is the second leading cause of death and the third leading cause of death and disability combined in 2019 (1). In China, stroke has become the top leading cause of death at the national level (2). In the past three decades, the epidemiologic characteristics and attributable risk factors of stroke in China have changed a lot (3). Ischemic stroke accounts for over 80% of all stroke cases (4). In 2019 alone, there were 2.87 million (95% uncertainty interval 2.38–3.46) new ischemic stroke cases in China (3). However, to date, a specific quantitative analysis of the time trends and gender differences of ischemic stroke in Chinese population is not available. Although several studies have investigated some respects of burden and risk factors of ischemic stroke in China, deeper analyses on the changes of epidemiologic characteristics and attributable risk factors are still needed (3, 5, 6).

The Global Burden of Disease (GBD) study of 2019 is the latest database that reports global estimates of various diseases (7). It provides an important tool for researchers to analyze disease burden and attributable risk factors around the world. A GBD 2019 stroke analysis showed that the age-standardized incidence rate of all stroke types in China decreased by 9.3%, whereas the age-standardized incidence rate of ischemic stroke increased by 34.7% (3). The disease burden of ischemic stroke is still severe in China. In this study, we investigated the time trends and gender differences of ischemic stroke burden and attributable risk factors in China between 1990 and 2019 based on the GBD study 2019. The burden and risk factors of ischemic stroke in China were compared to that of other countries. Differences in epidemiologic characteristics and attributable risk factors between men and women were observed. Results of this study will provide a reference for Chinese health policy makers to perfect public health policy of ischemic stroke prevention and control in the future.

## 2. Methods

### 2.1. Overview

The GBD study 2019 was designed to present epidemiological data and associated risk factors of diseases over time from 1990 to 2019 (7). The GBD database utilizes systematic or hierarchical sampling, ensuring equal chance of selection. The proportion of the population covered depends on data source availability and quality for each subgroup. The trends of disease burden of ischemic stroke in China were estimated using the following standard epidemiological measures: deaths, disability-adjusted life-years (DALYs), years lived with disability (YLDs), years of life lost (YLLs), prevalence, and incidence. Methods of extracting data from the GBD study have been described elsewhere (7). The 95% uncertainty intervals were determined by extracting the 25th and 975th values from the ordered set of values obtained from 1,000 posterior draws (8). This study was performed in accordance with the Guidelines for Accurate and Transparent Health Estimates Reporting (GATHER) (9). Ethical approval was not required as this study was based on publicly available data (GBD 2019) and no personal data was collected.

### 2.2. Case definitions

According to WHO criteria, stroke was defined as rapidly developing clinical signs of disturbance of cerebral function lasting more than 24 h or leading to death (10). In GBD study 2019, stroke was classified into three subcategories, consisting of ischemic stroke, intracerebral hemorrhage, and subarachnoid hemorrhage (3). Ischemic stroke was defined as an episode of neurological dysfunction due to limited blood flow to brain tissue and subsequent infarction (11).

### 2.3. Measures of burden

The measures of disease burden include deaths, DALYs, YLDs, YLLs, prevalence, and incidence of ischemic stroke by sex and age from 1990 to 2019. The Cause of Death Ensemble modelling (CODEm) method was used to estimate ischemic stroke-specific deaths (12). DALYs were used as common indicators to measure the disease burden of specific causes, consisting of the burden caused by YLDs and YYLs. YLDs were calculated as the prevalence of ischemic stroke multiplied by the corresponding disability weights (13). YLLs were calculated as the number of deaths cause by ischemic stroke multiplied by the remaining standard life expectancy at the age of death (14). Prevalence was defined as the number of newly confirmed cases and previous cases during the study period (3). Incidence was defined as the occurrence of first-ever ischemic stroke according to the criteria mentioned above (1). DisMod-MR 2.1, a Bayesian meta-regression modelling tool, was used to estimate the prevalence and incidence of ischemic stroke (15).

### 2.4. Risk factor estimation

Exposure to risks that exceeding the theoretical minimum risk exposure level (TMREL) was included to estimate risk factors due to ischemic stroke burden (16). Estimates of attributable risk factors were generated at the most detailed level. Risk factors included in this study consists of three series: environmental risks (ambient particulate matter pollution, household air pollution from solid fuels, high temperature, low temperature, and lead exposure), behavioral risks (smoking, secondhand smoke, alcohol use, and low physical activity), and metabolic risks (high systolic blood pressure, high low-density lipoprotein cholesterol (LDL), high fasting plasma glucose, high body-mass index (BMI), kidney dysfunction, diet high in sodium, diet high in red meat, diet low in whole grains, diet low in fruits, diet low in fiber, and diet low in vegetables). The risk factors were ranked according to the age-standardized ischemic stroke-related DALYs by year and sex (1). The correlations between disease deaths/DALYs rates and risk factors in corresponding years were analyzed using linear regression. Statistical analysis was performed using GraphPad Prism and a *p*-value of less than 0.05 was considered statistically significant.

### 2.5. Data presentation

Estimates were presented in terms of absolute numbers, rates per 100,000 people, and age-standardized rates per 100,000 people of deaths, DALYs, YLDs, YLLs, prevalence, and incidence (with 95% uncertainty intervals) and are stratified by sex. The age categories were all ages, age-standardized, under 5, 5–9, 10–14, 15–19, 20–24, 25–29, 30–34, 35–39, 40–44, 45–49, 50–54, 55–59, 60–64, 65–69, 70–74, 75–79, 80–84, 85–89, 90–94, and 95 plus years. Socio-demographic Index (SDI), is a comprehensive indicator that reflects the overall development level consisting of educational attainment, lagged distributed income, and total fertility rate (11). SDI were divided into five levels including high, high-middle, middle, low-middle, and low level. Burden and risk factors were compared between China and different SDI regions in this study. The World Bank divides the world’s economies into four income groups: high, upper middle, lower middle, and low income (17). The ischemic stroke burden of China was compared to countries with different incomes. Additionally, the burden and attributable risk factors of ischemic stroke in China was compared with similar big countries in the world (with large population and vast territory, including India, United States of America, and Brazil).

## 3. Results

### 3.1. Changes in the burden of ischemic stroke in China from 1990 to 2019

**Figure 1:**
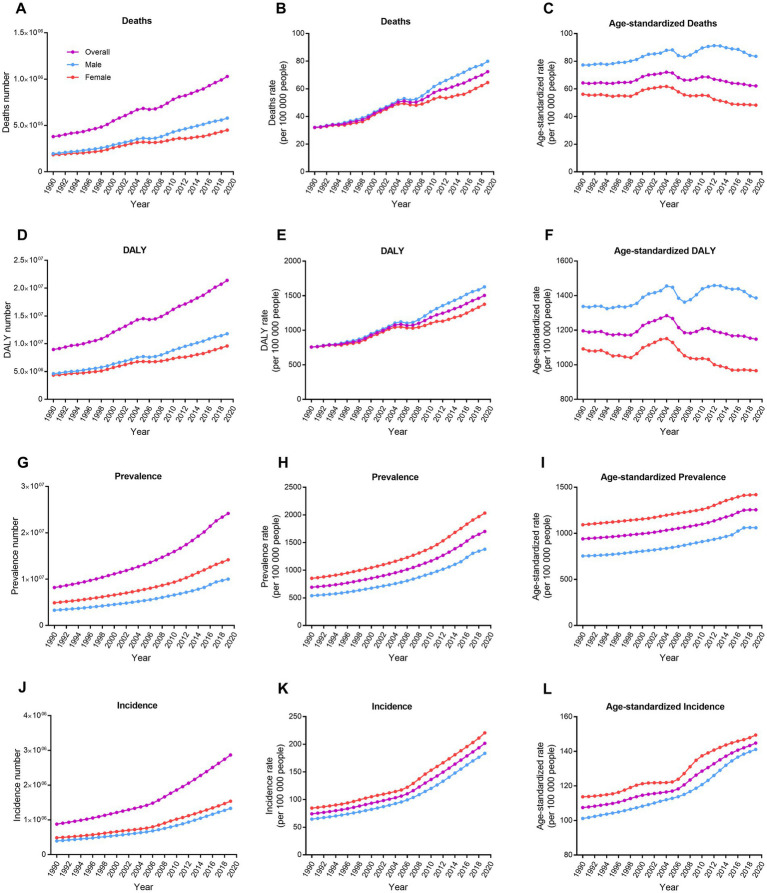
Trends in number, rate, and age-standardized rate for deaths, DALYs, prevalence, and incidence of ischemic stroke by sex in China from 1990 to 2019. **(A–C)** Deaths. **(D–F)** DALYs. **(G–I)** Prevalence. **(J–L)** Incidence. DALY, disability-adjusted life-year.

In China, there were 1.03 million (95% UI, 0.88–1.18) ischemic stroke induced-deaths, 21.4 million (95% UI, 18.7–24.4) DALYs due to ischemic stroke, 24.2 million (95% UI, 20.8–27.9) prevalent ischemic stroke cases, and 2.9 million (95% UI, 2.4–3.5) incident ischemic stroke cases in 2019 (Table 1; Figure 1). The age-standardized ischemic stroke death rates were 64.3 per 100,000 people (95% UI, 56.4–76.9 per 100,000 people) in 1990 and decreased to 62.2 per 100,000 people (95% UI, 53.3–70.7 per 100,000 people) in 2019, representing a 3.3% decrease over the past three decades. Age-standardized DALY rates of ischemic stroke decreased by 4%, from 1,196 per 100,000 people (95% UI, 1059.7–1402.8 per 100,000 people) to 1147.9 per 100,000 people (95% UI, 1008.6–1302.8 per 100,000 people). Age-standardized YLD rates increased by 35.1%, from 190.8 per 100,000 people (95% UI, 134.3–253.6 per 100,000 people) to 257.8 per 100,000 people (95% UI, 179.6–337.5 per 100,000 people; Supplementary Figure S1). Age-standardized YLL rates decreased by 11.4%, from 1005.2 per 100,000 people (95% UI, 876.9–1202.3 per 100,000 people) to 890.2 per 100,000 people (95% UI, 756.7–1023.6 per 100,000 people). Age-standardized prevalence rates of ischemic stroke were 940.7 per 100,000 people (95% UI, 814–1080.3 per 100,000 people) in 1990 and increased to 1255.9 per 100,000 people (95% UI, 1083.6–1437.6 per 100,000 people) in 2019, representing a 33.5% increase over the past 30 years. Age-standardized incidence rates increased by 34.7%, from 107.5 per 100,000 people (95% UI, 89.9–130.1 per 100,000 people) to 144.8 per 100,000 people (95% UI, 121.6–173.2 per 100,000 people; Table 1; Figure 1).

**Table 1:** Number, rate, and age-standardized rate for ischemic stroke deaths, disability-adjusted life-years, prevalence, and incidence in 2019 and percentage changes from 1990 and by sex in China.

	Deaths (95% UI)		DALYs (95% UI)		Prevalence (95% UI)		Incidence (95% UI)	
	2019	Percentage change, 1990–2019	2019	Percentage change, 1990–2019	2019	Percentage change, 1990–2019	2019	Percentage change, 1990–2019
Overall								
Number, in thousands	1,029 (881 to 1,176)	171.1% (109 to 228)	21,394 (18,721 to 24,376)	138.6% (94 to 181)	24,183 (20,804 to 27,869)	195.2% (174 to 219)	2,869 (2,376 to 3,459)	226.5% (211 to 243)
Rate, per 100,000	72.37 (61.97 to 82.7)	125.6% (74 to 173)	1504.12 (1316.2 to 1713.78)	98.6% (61 to 134)	1700.22 (1462.68 to 1959.39)	145.7% (128 to 165)	201.74 (167.03 to 243.17)	171.7% (159 to 185)
Age-standardized rate, per 100,000	62.18 (53.27 to 70.65)	−3.3% (−26 to 16)	1147.93 (1008.58 to 1302.75)	−4.0% (−23 to 13)	1255.91 (1083.57 to 1437.6)	33.5% (26 to 42)	144.78 (121.64 to 173.21)	34.7% (29 to 40)
Male								
Number, in thousands	579 (474 to 692)	195.7% (114 to 285)	11,795 (9,804 to 14,021)	155.6% (90 to 227)	10,001 (8,592 to 11,656)	203.5% (178 to 231)	1,331 (1,100 to 1,607)	237.7% (219 to 258)
Rate, per 100,000	79.89 (69.45 to 97.43)	149% (80 to 224)	1627.23 (1352.56 to 1934.4)	115.2% (60 to 175)	1379.82 (1185.34 to 1608.07)	155.5% (134 to 178)	183.6 (151.81 to 221.7)	184.3 (169 to 202)
Age-standardized rate, per 100,000	83.55 (69.45 to 97.43)	8% (−22 to 36)	1386.35 (1161.81 to 1624.31)	3.7% (−23 to 30)	1060.74 (911.73 to 1225.46)	40.6% (31 to 51)	141.1 (118.93 to 168.63)	39.5% (33 to 46)
Female								
Number, in thousands	450 (359 to 543)	144.9% (83 to 223)	9,599 (7,943 to 11,345)	120.7% (79 to 171)	14,182 (12,194 to 16,303)	189.7% (169 to 215)	1,539 (1,270 to 1871)	217.3 (199 to 236)
Rate, per 100,000	64.55 (51.47 to 77.95)	101.3 (51 to 165)	1376.19 (1138.72 to 1626.52)	81.4% (47 to 123)	2033.16 (1748.13 to 2337.21)	138.2% (121 to 159)	220.59 (182 to 268.27)	160.9 (146 to 176)
Age-standardized rate, per 100,000	48.26 (38.66 to 58.12)	−14% (−36 to 13)	965.78 (799.92 to 1135.34)	−11.5 (−29 to 9)	1419.3 (1223.57 to 1619.74)	29.8% (21 to 39)	149.44 (124.71 to 179.85)	31.4% (25 to 38)

### 3.2. Burden of ischemic stroke by sex and age

In China, the age-standardized ischemic stroke death rates were 83.6 per 100,000 people (95% UI, 69.5–97.4 per 100,000 people) in males and 48.3 per 100,000 people (95% UI, 38.7–58.1 per 100,000 people) in females (Table 1; Figure 1). Age-standardized death rates increased by 8% in males while decreased by 14% in females from 1990 to 2019. In 2019, the age-standardized DALY rates were 1386.4 per 100,000 people (95% UI, 1161.8–1624.3 per 100,000 people) in males and 965.8 per 100,000 people (95% UI, 799.9–1135.3 per 100,000 people) in females. Age-standardized DALY rates increased by 3.7% in males while decreased by 11.5% in females from 1990 to 2019. The age-standardized prevalence rates among males were 1060.7 per 100,000 people (95% UI, 911.7–1225.5 per 100,000 people) and 1419.3 per 100,000 people (95% UI, 1223.6–1619.7 per 100,000 people) among females. Age-standardized prevalence rates of ischemic stroke increased by 40.6% in males and 29.8% in females. Additionally, the age-standardized incidence rates among males were 141.1 per 100,000 people (95% UI, 118.9–168.6 per 100,000 people) and 149.4 per 100,000 people (95% UI, 124.7–179.9 per 100,000 people) among females. Age-standardized incidence rates of ischemic stroke increased by 39.5% in males and 31.4% in females (Table 1; Figure 1).

**Figure 2:**
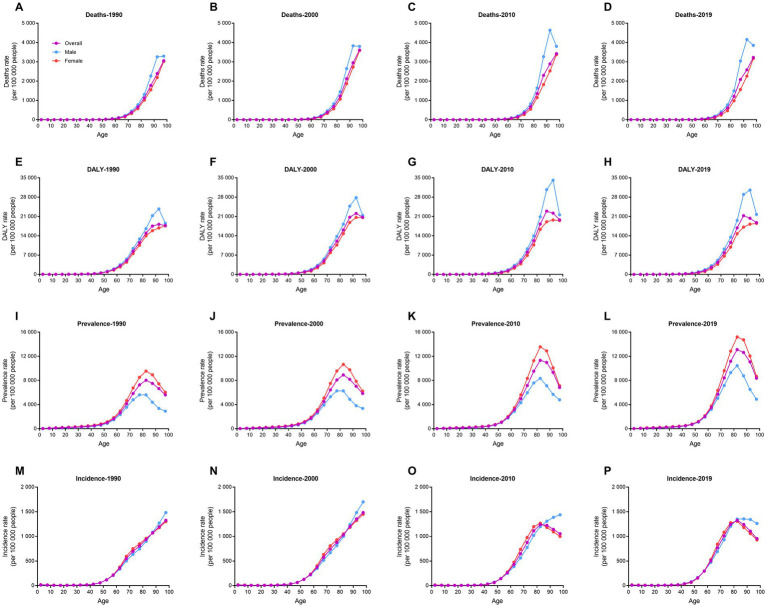
Trends in rate for deaths, DALYs, prevalence, and incidence of ischemic stroke in different age groups by sex in 1990, 2000, 2010, and 2019. **(A–D)** Deaths. **(E–H**) DALYs. **(I–L)** Prevalence. **(M–P)** Incidence. DALY, disability-adjusted life-year.

Death rates of ischemic stroke increased with age, especially markedly after 60 years old (Figure 2). DALY rates also increased with age, except for above 90 years old. In 2019, the highest DALY rate of ischemic stroke was in the 85–89 years of age (21294.4 per 100,000 people). After 50 years old, prevalence and incidence rates of ischemic stroke rose rapidly. The prevalence rates in Chinese elder population were significantly higher in 2019 compared to 1990. Incidence rates increased with age in both 1990 and 2000. Interestingly, the incidence rates reached their peaks in the 80–84 years of age in both 2010 and 2019 (1238.9 and 1326.4 per 100,000 people, respectively). The YLD rates in elder population were significantly higher in 2019 compared to 1990 (Supplementary Figure S2). YLL rates also increased with age, except for above 90 years old. Distinct gender differences were observed in all indicators.

### 3.3. Burden of ischemic stroke by SDI and income

**Figure 3:**
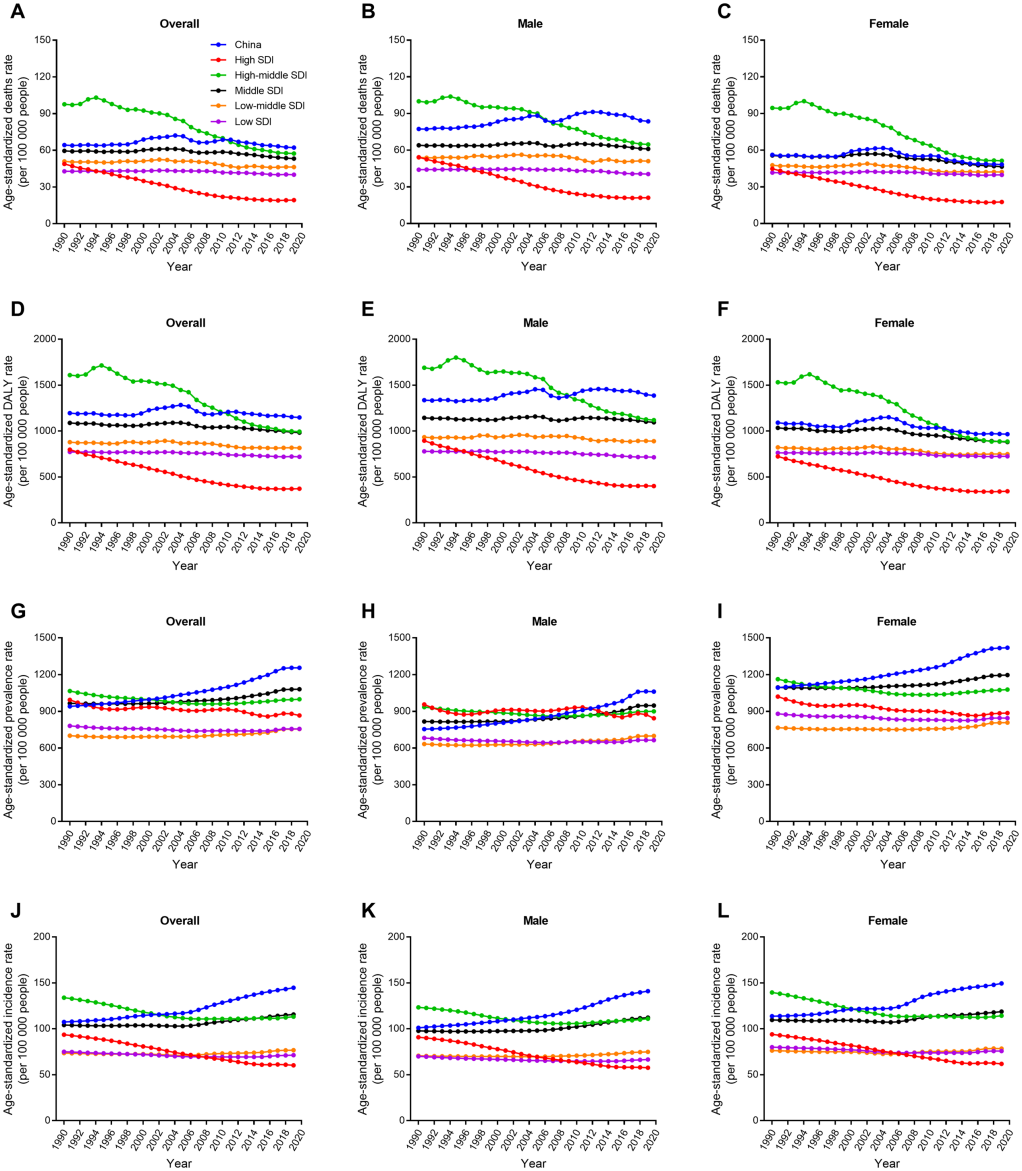
Trends in age-standardized rate for deaths, DALYs, prevalence, and incidence of ischemic stroke by sex in different SDI regions from 1990 to 2019. **(A–C)** Age-standardized deaths rate. **(D–F)** Age-standardized DALYs rate. **(G–I)** Age-standardized prevalence rate. **(J–L)** Age-standardized incidence rate. DALY, disability-adjusted life-year. SDI, socio-demographic index.

**Figure 4:**
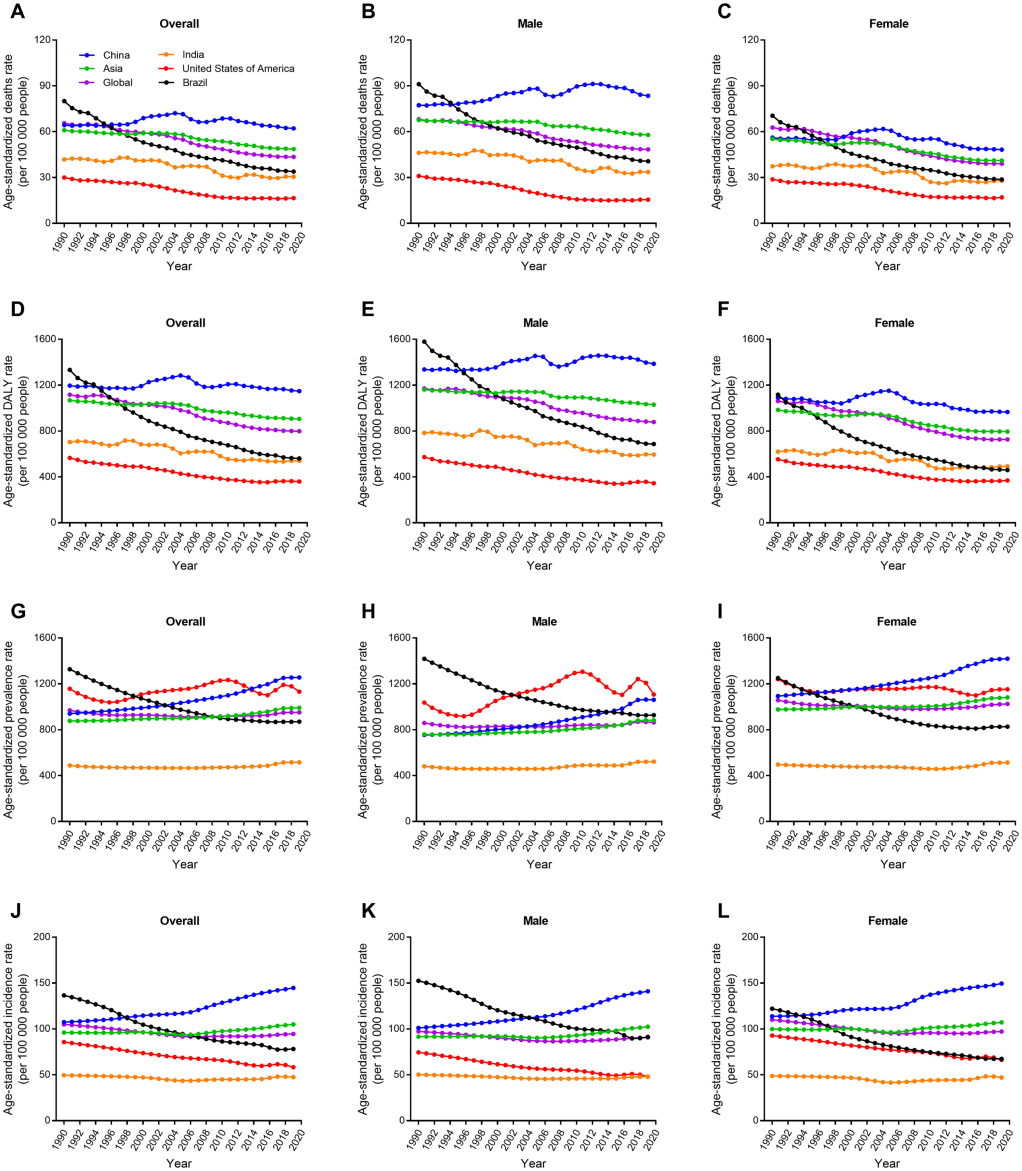
Trends in age-standardized rate for deaths, DALYs, prevalence, and incidence of ischemic stroke by sex in four big countries from 1990 to 2019. **(A–C)** Age-standardized deaths rate. **(D–F)** Age-standardized DALYs rate. **(G–I)** Age-standardized prevalence rate. **(J–L)** Age-standardized incidence rate. DALY, disability-adjusted life-year.

Globally, regions were divided into five groups according to the SDI value (18). The age-standardized death and DALY rates of ischemic stroke in high and high-middle SDI groups decreased markedly from 1990 to 2019 (−53.4% and-38.3%, respectively; Figure 3). However, age-standardized death and DALY rates in China and other SDI groups hardly changed in the past 30 years. China had the highest increase in age-standardized prevalence and incidence rates of ischemic stroke among all the groups (33.5 and 34.7%, respectively). Furthermore, China had the highest age-standardized death, DALY, prevalence, and incidence rates in 2019. World Bank classified global regions into four groups according to income levels: high, upper middle, lower middle, and low (19). Similar to above results, China had the highest age-standardized death, DALY, prevalence, and incidence rates of ischemic stroke among all the income groups in 2019 (Supplementary Figure S3). The disease burden of ischemic stroke in China was also compared with global and Asia levels, as well as with three similar countries with large population and territory (India, USA, and Brazil). The results showed that China still had the highest age-standardized death, DALY, prevalence, and incidence rates among the six groups (Figure 4). USA had the lowest age-standardized death and DALY rates and India had the lowest age-standardized prevalence and incidence rates in the past three decades.

### 3.4. Changes in risk factors of ischemic stroke in China from 1990 to 2019

**Figure 5:**
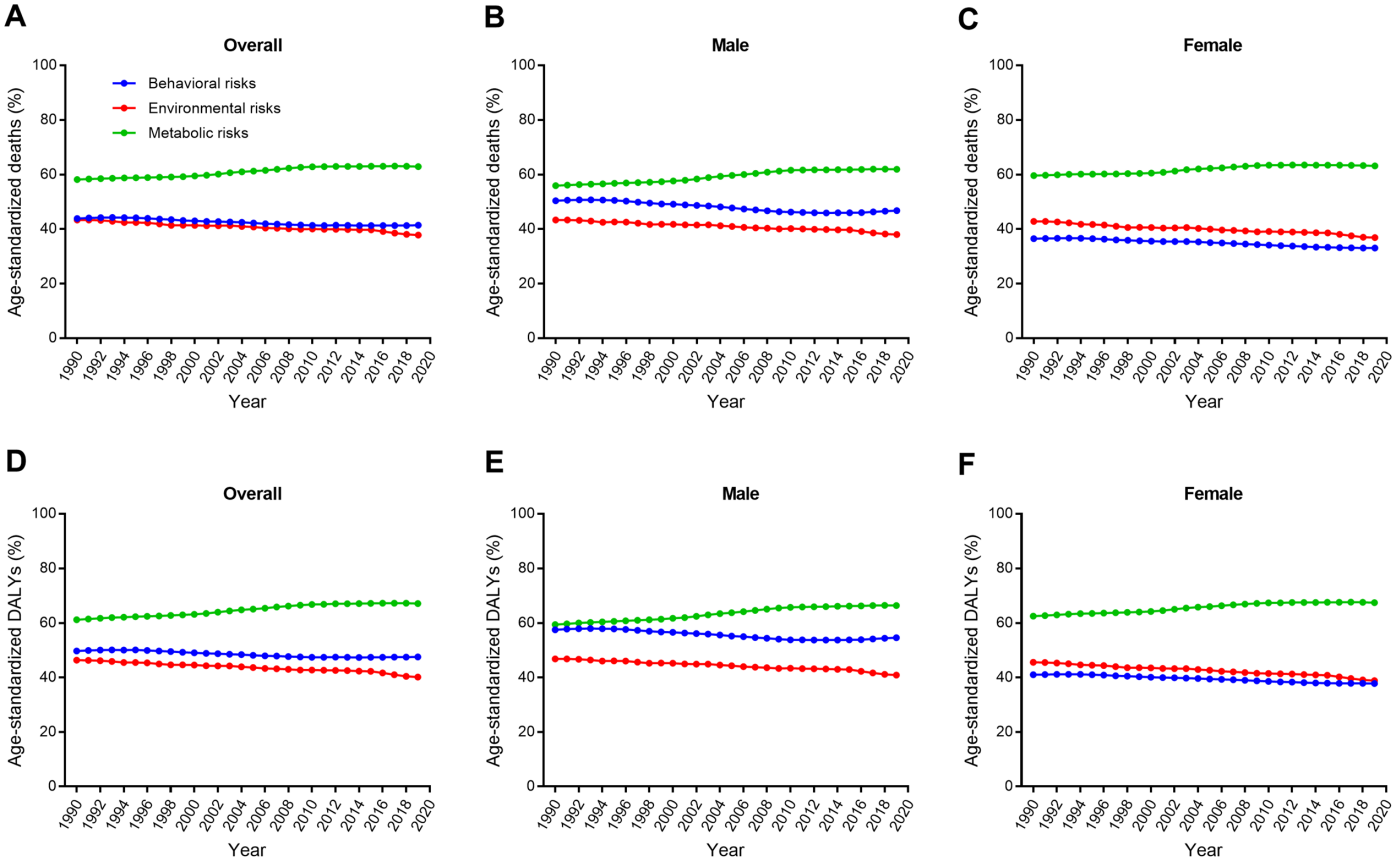
Trends in age-standardized ischemic stroke-related death and DALY percent for level one attributable risk factors by sex from 1990 to 2019. **(A–C)** Age-standardized death percent. **(D–F)** Age-standardized DALY percent. DALY, disability-adjusted life-year.

**Figure 6:**
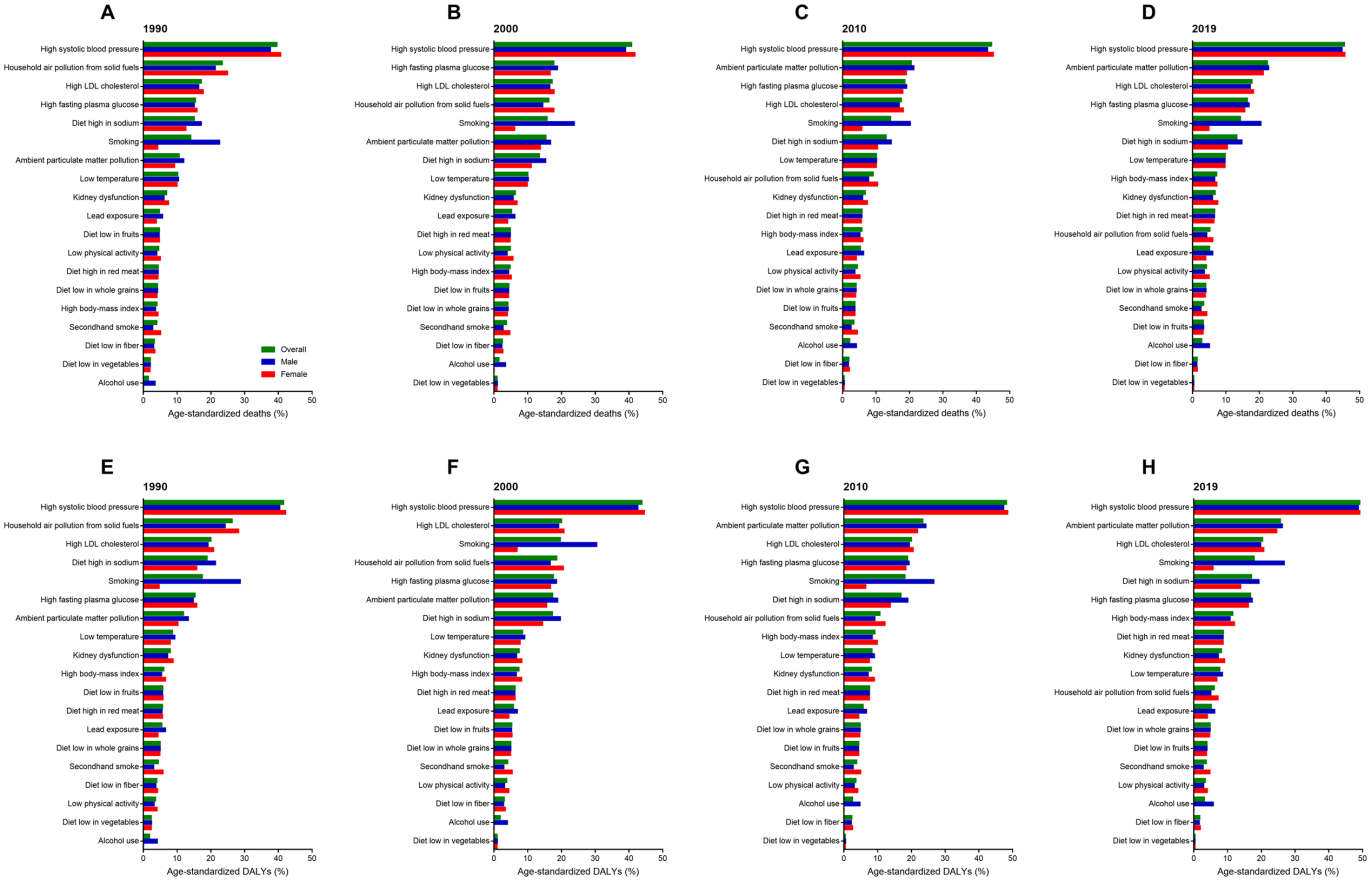
Attributable risk factors of age-standardized ischemic stroke-related death and DALY percent by sex in 1990, 2000, 2010, and 2019. **(A–D)** Age-standardized death percent. **(E–H)** Age-standardized DALY percent. DALY, disability-adjusted life-year.

**Figure 7:**
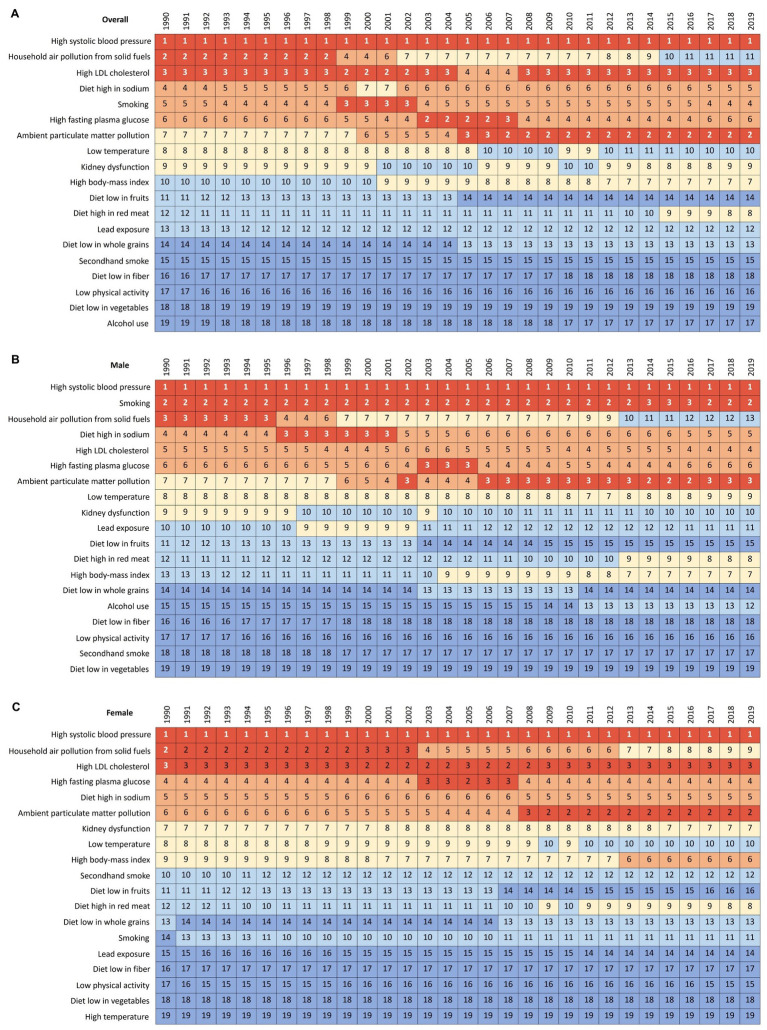
Changes in attributable risk factors of age-standardized ischemic stroke-related DALYs by year and sex from 1990 to 2019. **(A)** Both sexes. **(B)** Male. **(C)** Female. DALY, disability-adjusted life-year.

In China, metabolic risks were the first leading risk factors for ischemic stroke deaths and DALYs from 1990 to 2019 (Table 2; Figure 5). An interesting finding was that behavioral risks were the second leading risk factors for males but environmental risks were the second leading risk factors for females. From 1990 to 2019, detailed risk factors attributable to ischemic stroke deaths and DALYs had made profound changes. The first leading risk factor for ischemic stroke was high systolic blood pressure in the last three decades (Figure 6). Household air pollution from solid fuels was the second leading risk factors in 1990 and downed to 11th by 2019 (Figure 7). In contrast, ambient particulate matter pollution rose from 7th to 2th. Smoking was an important risk factor for ischemic stroke deaths and DALYs in males, but not a vital factor for females.

**Table 2:** Attributable age-standardized deaths and DALYs percent by ischemic stroke risk factors in 2019 and by sex in China.

	Age-standardized deaths percent (95% UI)			Age-standardized DALYs percent (95% UI)		
	Overall	Male	Female	Overall	Male	Female
Level 1 risks						
Environmental risks	38% (35 to 41)	38% (35 to 41)	37% (34 to 40)	40% (37 to 43)	41% (38 to 44)	39% (36 to 42)
Behavioral risks	41% (34 to 50)	47% (39 to 55)	33% (24 to 43)	48% (39 to 56)	55% (47 to 62)	38% (29 to 47)
Metabolic risks	63% (50 to 76)	62% (49 to 75)	63% (50 to 77)	67% (57 to 77)	66% (55 to 76)	67% (57 to 78)
Environmental risks						
Ambient particulate matter pollution	22.3% (19 to 25)	22.7% (19.6 to 25.2)	21.1% (17.6 to 23.9)	25.6% (21.8 to 28.5)	26.2% (22.7 to 28.9)	24.5% (20.4 to 27.6)
Household air pollution from solid fuels	5.1% (2.6 to 8.8)	4.2% (2 to 7.6)	6% (3.2 to 9.7)	6% (3.1 to 10.2)	5% (2.3 to 8.9)	7.1% (3.8 to 11.4)
High temperature	0.1% (−0.1 to 0.5)	0.1% (−0.1 to 0.5)	0.1% (−0.1 to 0.5)	0.1% (−0.1 to 0.4)	0.1% (−0.1 to 0.4)	0.1% (−0.1 to 0.3)
Low temperature	9.7% (7.7 to 12.2)	9.7% (7.7 to 12.2)	9.7% (7.7 to 12.1)	7.7% (6 to 9.8)	8.4% (6.5 to 10.8)	6.8% (5.2 to 8.7)
Lead exposure	5.1% (3 to 7.5)	6% (3.7 to 8.7)	3.9% (2.2 to 6.1)	5.2% (3.2 to 7.4)	6.2% (4 to 8.5)	4% (2.2 to 6)
Behavioral risks						
Smoking	14.3% (12.8 to 16)	20.4% (19 to 21.8)	4.8% (4.1 to 5.7)	17.8% (16.2 to 19.5)	26.8% (25.3 to 28.4)	5.6% (4.8 to 6.5)
Secondhand smoke	3.3% (2.5 to 4.2)	2.5% (1.8 to 3.3)	4.2% (3.2 to 5.4)	3.6% (2.7 to 4.6)	2.7% (2 to 3.5)	4.7% (3.5 to 6)
Alcohol use	2.7% (0.7 to 4.7)	5% (1.5 to 8.8)	−0.1% (−0.8 to 0.7)	3.1% (0.9 to 5.3)	5.7% (1.8 to 9.8)	−0.1% (−0.8 to 0.6)
Low physical activity	4.1% (0.7 to 11.6)	3.5% (0.4 to 10.8)	4.9% (0.9 to 12.9)	3.3% (0.6 to 9.5)	2.9% (0.4 to 8.8)	3.9% (0.7 to 10.5)
Metabolic risks						
High systolic blood pressure	45.4% (33.3 to 58.1)	44.6% (32 to 58)	45.4% (32.2 to 60.1)	49.2% (38.1 to 59.7)	48.7% (36.7 to 59.7)	49.1% (36.5 to 60.8)
High LDL cholesterol	17.7% (5.9 to 37.9)	17.2% (5.7 to 36.8)	18.2% (5.5 to 39.6)	20.3% (10.7 to 36.2)	19.7% (10.4 to 35.4)	20.7% (10.7 to 37.2)
High fasting plasma glucose	16.4% (8.1 to 34)	16.8% (8.1 to 35.2)	15.6% (7.6 to 33.7)	16.7% (9 to 30.2)	17.2% (9.1 to 31.8)	16.1% (8.9 to 29.7)
High body-mass index	7.1% (3 to 12.6)	6.6% (2.6 to 11.9)	7.2% (3.1 to 12.8)	11.5% (5.3 to 18.8)	10.7% (4.8 to 17.7)	12% (5.7 to 19.6)
Kidney dysfunction	6.8% (4.3 to 9.2)	5.9% (3.6 to 8.3)	7.5% (4.7 to 10.2)	8.1% (6 to 10.3)	7.2% (5.2 to 9.1)	9.1% (6.8 to 11.5)
Diet high in sodium	13.2% (4.4 to 24.9)	14.7% (5.4 to 26.8)	10.4% (2.4 to 22.1)	17% (6.8 to 29.2)	19.2% (8.1 to 31.4)	13.8% (4.2 to 26.2)
Diet high in red meat	6.6% (3.3 to 8.8)	6.5% (3.1 to 8.9)	6.4% (3.1 to 8.8)	8.7% (4.7 to 11.3)	8.6% (4.5 to 11.3)	8.6% (4.7 to 11.3)
Diet low in whole grains	3.9% (1 to 5.8)	4% (1 to 5.9)	3.8% (1 to 5.6)	4.8% (1.2 to 7.1)	4.8% (1.2 to 7.1)	4.6% (1.2 to 6.9)
Diet low in fruits	3.2% (0.8 to 6.1)	3.3% (0.9 to 6.2)	3.1% (0.8 to 6)	3.8% (0.9 to 7.3)	3.9% (1 to 7.3)	3.7% (0.9 to 7.1)
Diet low in fiber	1.3% (0.4 to 2.7)	1.3% (0.4 to 2.6)	1.4% (0.4 to 2.9)	1.7% (0.4 to 3.4)	1.6% (0.4 to 3.2)	1.8% (0.5 to 3.7)
Diet low in vegetables	0.3% (0.2 to 0.5)	0.3% (0.2 to 0.6)	0.3% (0.2 to 0.5)	0.3% (0.2 to 0.4)	0.3% (0.2 to 0.5)	0.3% (0.2 to 0.4)

**Figure 8:**
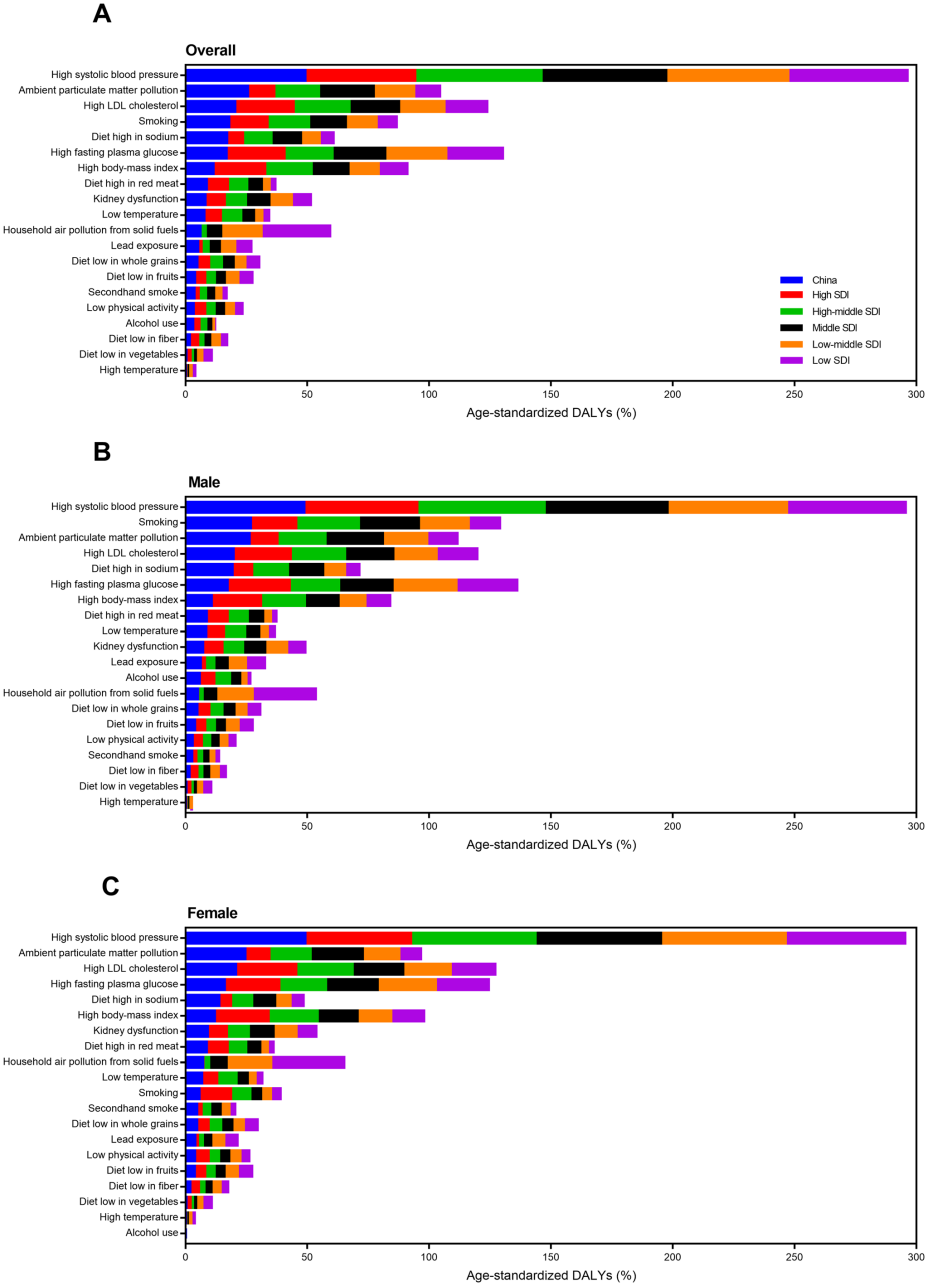
Attributable risk factors of age-standardized ischemic stroke-related DALY percent in different SDI regions by sex in 2019. **(A)** Both sexes. **(B)** Male. **(C)** Female. DALY, disability-adjusted life-year. SDI, socio-demographic index.

**Figure 9:**
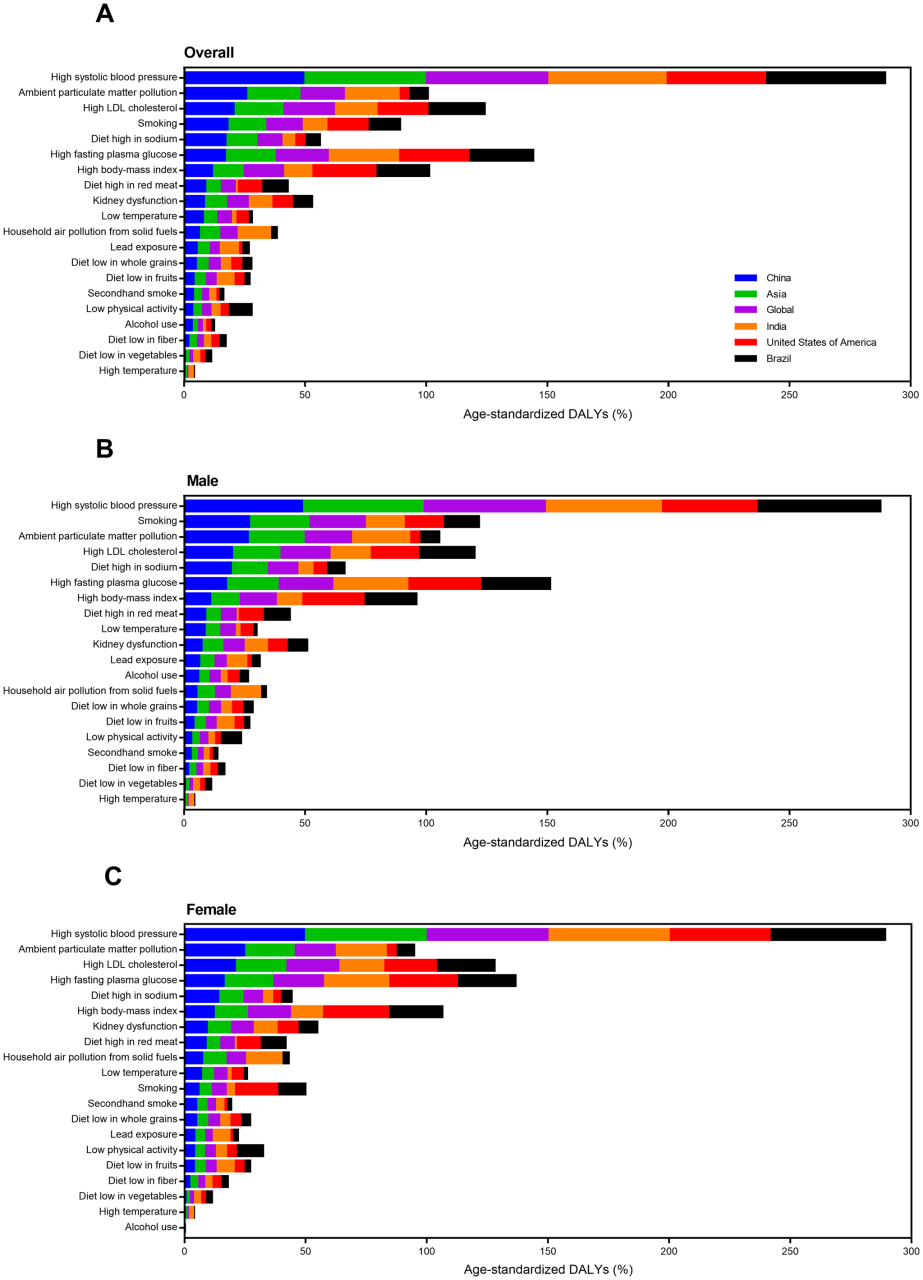
Attributable risk factors of age-standardized ischemic stroke-related DALY percent in four big countries by sex in 2019. **(A)** Both sexes. **(B)** Male. **(C)** Female. DALY, disability-adjusted life-year.

High systolic blood pressure was a common risk factor for all SDI groups. In 2019, high BMI was less important risk factors, and ambient particulate matter pollution and diet high in sodium were more vital factors for China compared to high SDI group (Figure 8). Compared to low SDI group, household air pollution from solid fuels was less important, and ambient particulate matter pollution, smoking, and diet high in sodium were more important risk factors for China. For USA, high fasting plasma glucose and high BMI were more important for ischemic stroke DALYs (Figure 9). For India, high fasting plasma glucose was the second leading risk factor for ischemic stroke. For Brail, ambient particulate matter pollution and diet high in sodium were not important but high fasting plasma glucose and high BMI were very important risk factors for ischemic stroke. Gender difference was also observed here. For example, smoking was a more important risk that inducing ischemic stroke for males in China than the other three countries.

### 3.5. Correlation analysis

**Figure 10:**
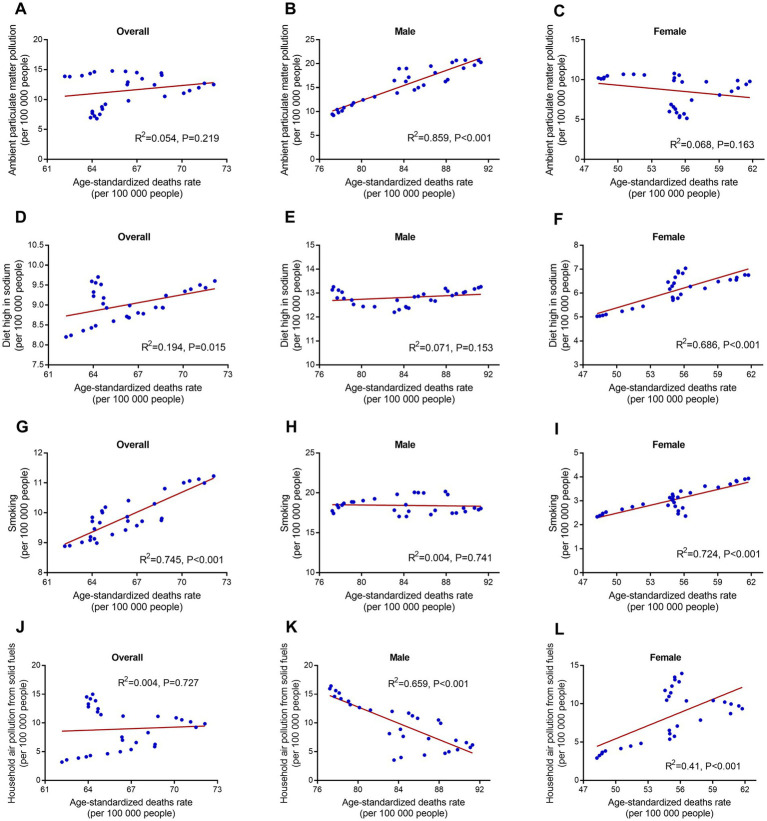
linear regression of risk factors and age-standardized ischemic stroke-related deaths rate in corresponding years by sex from 1990 to 2019. **(A–C)** Ambient particulate matter pollution. **(D–F)** Diet high in sodium. **(G–I)** Smoking. **(J–L)** Household air pollution from solid fuels.

The correlations between age-standardized deaths/DALYs and risk factors in corresponding years were analyzed in this study. Ambient particulate matter pollution was associated with ischemic stroke-induced deaths rate increase in males (*R^2^* = 0.859, *p* < 0.001), but not associated with females (*R^2^* = 0.068, *p* = 0.163) in the past 30 years in China (Figure 10). Diet high in sodium and smoking were both associated with increase of death rates due to ischemic stroke in females (*R^2^* = 0.686, *p* < 0.001, and *R^2^* = 0.724, *p* < 0.001, respectively), but not males (*R^2^* = 0.071, *p* = 0.153, and *R^2^* = 0.004, *p* = 0.741, respectively). Household air pollution from solid fuels had significantly negative correlation with ischemic stroke-caused death rates increase in males (*R^2^* = 0.659, *p* < 0.001), but had significantly positive correlation with females (*R^2^* = 0.41, *p* < 0.001). Ambient particulate matter pollution, diet high in sodium, smoking, and household air pollution from solid fuels had similar gender association with ischemic stroke-caused DALY rates (Supplementary Figure S4). Additionally, high fasting plasma glucose, high LDL cholesterol, and high systolic blood pressure were all associated with ischemic stroke-specific death/DALY rates increase, regardless of males and females (Supplementary Figures S5, S6).

## 4. Discussion

To our knowledge, this is the first study that comprehensively analyzed the disease burden and attributable risk factors of ischemic stroke in China from 1990 to 2019 base on the GBD study 2019.

In this study, the changes in burden and risk factors of ischemic stroke from 1990 to 2019 were described at the national level of China. The changes in China were compared to regions with different SDI and income in the world. Our results showed China has faced new challenges on ischemic stroke preventing and controlling.

In the past three decades, the absolute number of deaths, DALYs, YLDs, YLLs, prevalence, and incidence due to ischemic stroke increased substantially in China. The large increase may be explained by the changes in the demographic structure such as ageing and population growth (1). However, age-standardized death and DALY rates of ischemic stroke did not change significantly during the last 30 years. The age-standardized ischemic stroke death and DALY rates even decreased by 3.3 and 4%, respectively. The advancements were probably attributed to significant improvements of living condition and medical care in China over the past three decades (20). The age-standardized prevalence and incidence rates increased markedly, which may be partly due to improvement in diagnostic level and increase in unhealthy life styles (e.g., smoking, diet high in red meat, high BMI) (21).

Gender is an important factor influencing the disease burden of ischemic stroke (22). The age-standardized death and DALY rates due to ischemic stroke were greater in males than in females, which suggests that ischemic stroke is more harmful to men than women. The results of YLD and YLL also suggest that men are more likely to die and women are more likely to disability after attacked by ischemic stroke. This is probably because men are more likely than women to have unhealthy lifestyles, such as smoke and drink (23). Moreover, higher level of estrogen in females might also contribute to this situation because of the neuroprotective effects of estrogen (24). Interestingly, we found that age-standardized prevalence and incidence rates in males were lower than in females, suggesting that women are more likely to have ischemic stroke than men. This finding contradicts results from previous studies (25, 26). Racial disparity and age variability might probably explain this paradox.

It is well known that age is a major risk factor for ischemic stroke (27). The overall death rate of ischemic stroke in China is increasing with age, especially for elderly over 70 years old. However, we found that death rate among males decreased over 95 years old after 2000. That may be because men over 95 years old are more likely to suffer from other fatal diseases (26). The incidence rate of ischemic stroke increased with age in 1990 and 2000, but decreased with age in people over 85 years old in 2019 (especially in females). The incidence rate among females was higher than males between 60 to 80 years old. But after 80 years old, incidence rate among males was higher than females. This finding is different from previous study (28). Further study is needed to investigate the sex difference of ischemic stroke burden among very old people. The prevalence rate among females was significantly higher than males over 60 years old. One possible explanation is that estrogen in women decreases rapidly after menopause (24). Higher death rate in males may be also accountable for this situation. In addition, the changes in DALY rate attributable to ischemic stroke are largely determined by YLL rate.

SDI reflects the comprehensive level of social development. Globally, increase in SDI accompanied by decrease in stroke burden over time (11). The ischemic stroke burden of China were the highest among all the SDI groups in 2019, suggesting the serious situation faced by China. The high deaths and DALYs of ischemic stroke in China were mostly due to the high deaths and DALYs among Chinese males. Additionally, China has the fastest growth rates in incidence and prevalence of ischemic stroke during the past 30 years. Many factors such as rapid economic development, increased environmental pollution, and intensified aging population may be involved (29, 30). The burden of ischemic stroke in China were still the highest among all groups with different income levels. China, USA, India, and Brazil are highly representative large countries in terms of geographical coverage and population, making them comparable on a global scale. Compared to these countries, the burden of ischemic stroke in China were still the highest. Among the three countries, USA has the lowest death and DALY and India has the lowest incidence and prevalence. There are still huge gaps in the prevention and controlling of ischemic stroke for China despite great progress has been made.

Metabolic risks were the most important risk factors attributable to ischemic stroke in China (31, 32). Behavioral risks were the second most important risk factors among males and environmental risks were the second most important risk factors among females. The reason may be that men smoke and drink significant more than women (33). High systolic blood pressure remained the first leading risk factor for ischemic stroke in the past 30 years (34). However, other risk factors have changed a lot over time. Household air pollution from solid fuels used to be a vital risk for ischemic stroke but it became unimportant now. The Chinese government’s efforts to expand the use of clean fuels (e.g., marsh gas and solar energy) might account for this change (35). Furthermore, ambient particulate matter pollution has become the second leading risk for ischemic stroke in China (36). Rapid growth of motor vehicles and increased urbanization in China during the past two decades might lead to this result (37, 38). Smoking remained the second important risk factor after hypertension for males in China (39). Diet high in red meat and high BMI are becoming increasingly important risks for ischemic stroke. Unhealthy eating habits and overweight are becoming more common in Chinese population, which should catch our attention (40).

Risk factors attributable to ischemic stroke distinctly differ in regions with different SDI levels. High BMI was an important risk for ischemic stroke in high SDI regions in 2019. Whereas in low SDI regions, high BMI was less important and household air pollution from solid fuels remained a major risk. In China, differed with them, ambient particulate matter pollution and diet high in sodium were vital risks for ischemic stroke. The main cause of China’s growing environmental pollution is the sharp rise in energy consumption that accompanied rapid development in the past 30 years (41). Sodium intake is high in China may be due to the high-salt eating habits of Chinese population (42). Among big countries, ambient particulate matter pollution was an important risk in China and India. Rapid economic development in these two countries in the past three decades might account for this (43). For USA and Brazil, high BMI was a more important risk attributable to ischemic stroke. In India, household air pollution from solid fuels was more severe and the diet in red meat was significantly less. This was probably associated with the life style and religious custom of Indians.

We also analyzed the correlation between deaths/DALYs and risk factors in corresponding year. This indicator reflects whether a specific risk was involved in increased burden of ischemic stroke during the past 30 years in China. We found that ambient particulate matter pollution was associated with increase of deaths/DALYs in males rather than in females. This result was differed from previous studies and should be studied further (44–46). Diet high in sodium among females rather than males was associated with deaths/DALYs increase. This may be because that women are more salt-sensitive than men (47). Interestingly, we found that smoking in females rather than in males was relevant to increased deaths/DALYs due to ischemic stroke. This result, in agreement with previous studies, that women are more susceptible to smoking effects than men (48, 49). Household air pollution from solid fuels was more relevant with increased deaths/DALYs in females rather than in males. This might be partly because that women cook more frequently than man (50, 51).

Many similar studies have been published to study the disease burden of stroke based on the GBD database. A lot of studies have analyzed the global burden of stroke and its risk factors (1, 11, 52). At the national level of China, the burden of stroke has also been analyzed systematically by many researchers (3, 5, 6, 53, 54). In the aspect of ischemic stroke, the global burden of ischemic stroke has been well studied (55–57). However, the disease burden and risk factors of ischemic stroke in China has not been comprehensively analyzed. To the best of our knowledge, this study is the first one to comprehensively analyze the burden and attributable risk factor of ischemic stroke in China base on GBD study.

## 5. Limitations

This study has several limitations. First, estimates in this study was generated using GBD data and methodologies. Although data from GBD database is generally considered to be reliable, audience should also be aware of the general limitations of GBD study (58, 59). Second, we were not able to provide analysis on epidemiologic changes of ischemic stroke at provincial level in China due to the limited data in GBD study 2019. Third, analyses on the ischemic stroke subtypes (e.g., large artery atherosclerosis, small vessel occlusion, cardioembolism, other determined aetiology, and undetermined aetiology) (60) were unable to conduct because the GBD framework did not classify ischemic stroke. Despite these limitations, this study provides useful information for us to know the current status of ischemic stroke burden and risk factors in China using available data.

## 6. Conclusion

The present study revealed that the disease burden of ischemic stroke in China is still severe. There is much room for improvement in terms of ischemic stroke preventing and controlling situation in China, compared with other countries in the world, especially with developed countries. Distinct gender differences in disease burden and risk factors were observed during the 30-year observation period. Risk factors for ischemic stroke in China had changed a lot from 1990 to 2019. Public health policy should be adjusted accordingly with the changed situation to better reduce the burden of ischemic stroke.

## Data availability statement

The original contributions presented in the study are included in the article/Supplementary material, further inquiries can be directed to the corresponding author.

## Author contributions

YY, X-YX, and DL conceived this study. YY and Y-TZ accessed and acquired the raw data, performed the primary analysis, prepared tables and figures, and drafted the first manuscript. J-CZ, H-LZ, R-WF, Y-XJ, and H-QH contributed to the interpretation of the data. X-YX critically revised the manuscript. All authors reviewed the article, read the final manuscript and approved the submission.

## Funding

This study was supported by the National Natural Science Foundation of China (grant nos. 82074193 and 81903942) and Peking University Medicine Seed Fund for Interdisciplinary Research (grant no. BMU2018MX027).

## Conflict of interest

The authors declare that the research was conducted in the absence of any commercial or financial relationships that could be construed as a potential conflict of interest.

## Publisher’s note

All claims expressed in this article are solely those of the authors and do not necessarily represent those of their affiliated organizations, or those of the publisher, the editors and the reviewers. Any product that may be evaluated in this article, or claim that may be made by its manufacturer, is not guaranteed or endorsed by the publisher.
