# Supplementary material for: Burden and attributable risk factors of ischemic stroke in China from 1990 to 2019: an analysis from the Global Burden of Disease Study 2019
Source: Front Neurol. 2023 Jul 26;14:1216777. doi: 10.3389/fneur.2023.1216777 (PMC10409994; doi:10.3389/fneur.2023.1216777)
Supplement: Supplementary file 1 [file Data_Sheet_1.PDF]

Supplementary Materials

Supplementary Figure 1. Trends in number, rate, and age-standardized rate for YLDs and YLLs of ischemic stroke by sex in China from 1990 to 2019. (A-C) YLDs. (D-F) YLLs. YLDs, years lived with disability. YLLs, years of life lost.

Supplementary Figure 2. Trends in rate for YLDs and YLLs of ischemic stroke in different age groups by sex in 1990, 2000, 2010, and 2019. (A-D) YLDs. (E-H) YLLs. YLDs, years lived with disability. YLLs, years of life lost.

Supplementary Figure 3. Trends in age-standardized rate for deaths and DALYs of ischemic stroke by sex in different income groups from 1990 to 2019. (A-C) Age-standardized deaths rate. (D-F) Age-standardized DALYs rate. (G-I) Age-standardized prevalence rate. (J-K) Age-standardized incidence rate. DALY, disability-adjusted life-year.

Supplementary Figure 4. linear regression of risk factors and age-standardized ischemic stroke-related DALYs rate in corresponding years by sex from 1990 to 2019. (A-C) Ambient particulate matter pollution. (D-F) Diet high in sodium. (G-I) Smoking. (J-L) Household air pollution from solid fuels. DALY, disability-adjusted life-year.

Supplementary Figure 5. linear regression of risk factors and age-standardized ischemic stroke-related deaths rate in corresponding years by sex from 1990 to 2019. (A-C) High fasting plasma glucose. (D-F) High LDL cholesterol. (G-I) High systolic blood pressure. LDL, low-density lipoprotein.

Supplementary Figure 6. linear regression of risk factors and age-standardized ischemic stroke-related DALYs rate in corresponding years by sex from 1990 to 2019. (A-C) High fasting plasma glucose. (D-F) High LDL cholesterol. (G-I) High systolic blood pressure. DALY, disability-adjusted life-year. LDL, low-density lipoprotein.

Supplementary Figure 1

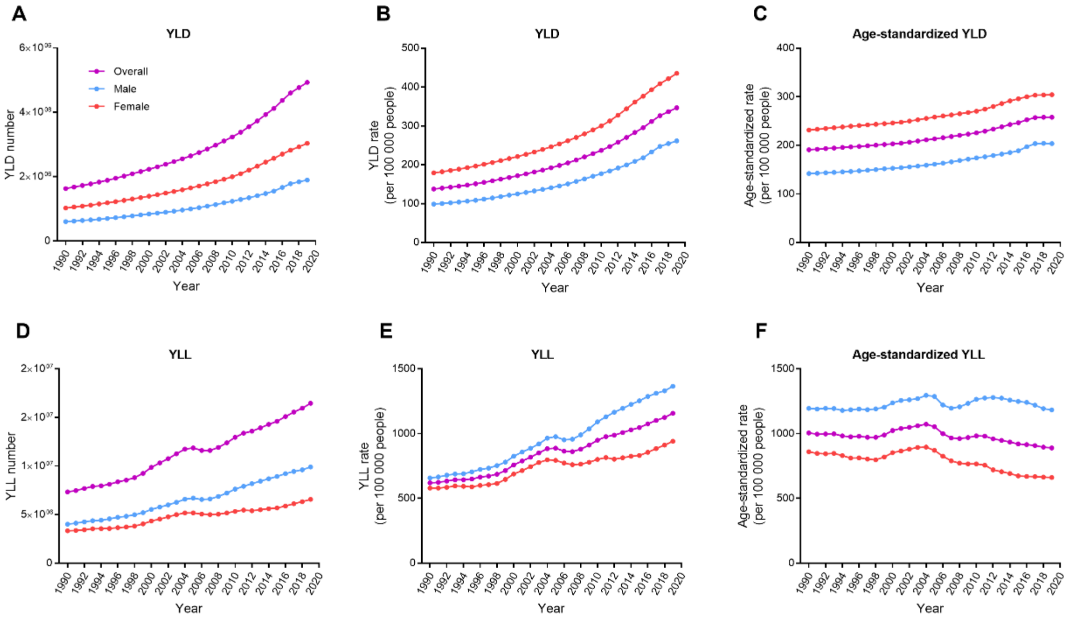

Figure S1. Trends in number, rate, and age-standardized rate for YLDs and YLLs of ischemic stroke by sex in China from 1990 to 2019. (A-C) YLDs. (D-F) YLLs. YLDs, years lived with disability. YLLs, years of life lost.

Supplementary Figure 2

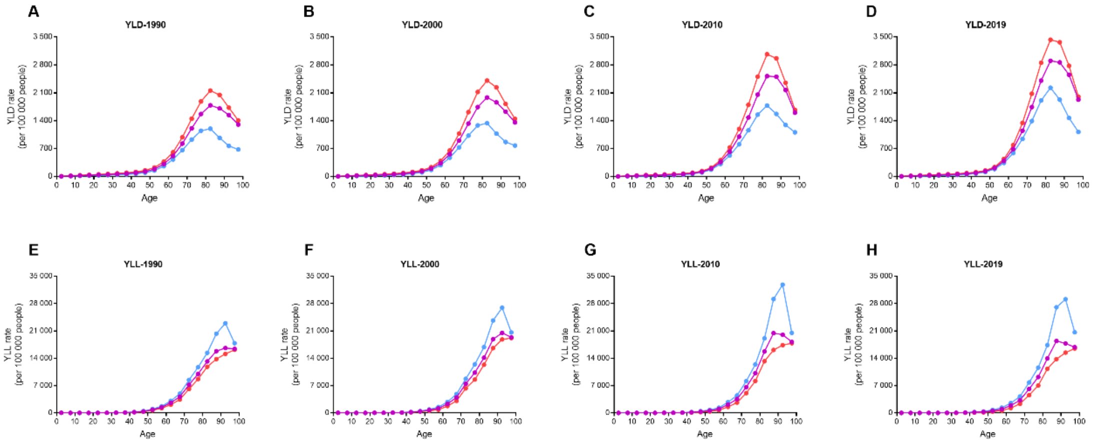

Figure S2. Trends in rate for YLDs and YLLs of ischemic stroke in different age groups by sex in 1990, 2000, 2010, and 2019. (A-D) YLDs. (E-H) YLLs. YLDs, years lived with disability. YLLs, years of life lost.

Supplementary Figure 3

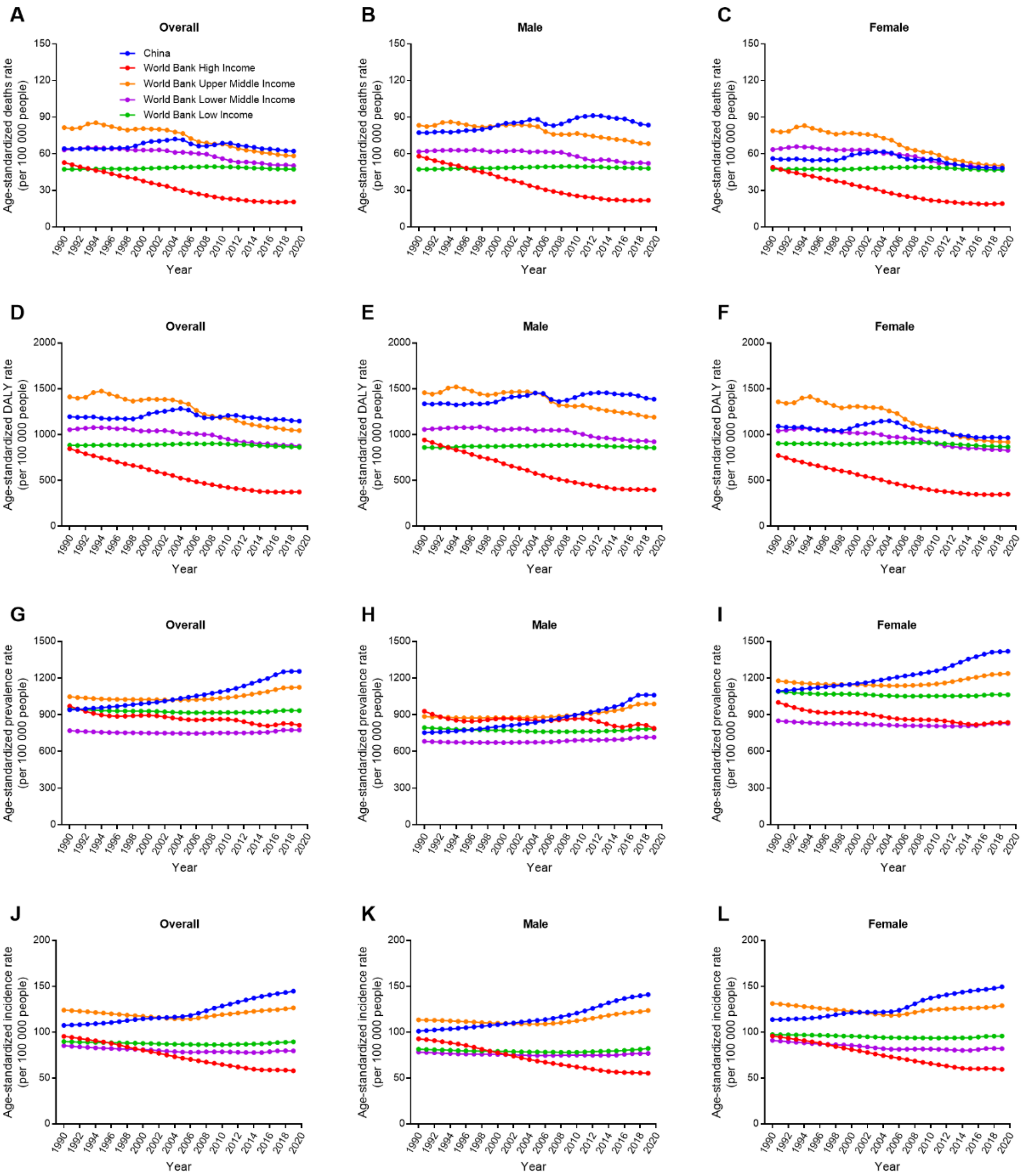

Figure S3. Trends in age-standardized rate for deaths and DALYs of ischemic stroke by sex in different income groups from 1990 to 2019. (A-C) Age-standardized deaths rate. (D-F) Age-standardized DALYs rate. (G-I) Age-standardized prevalence rate. (J-K) Age-standardized incidence rate. DALY, disability-adjusted life-year.

Supplementary Figure 4

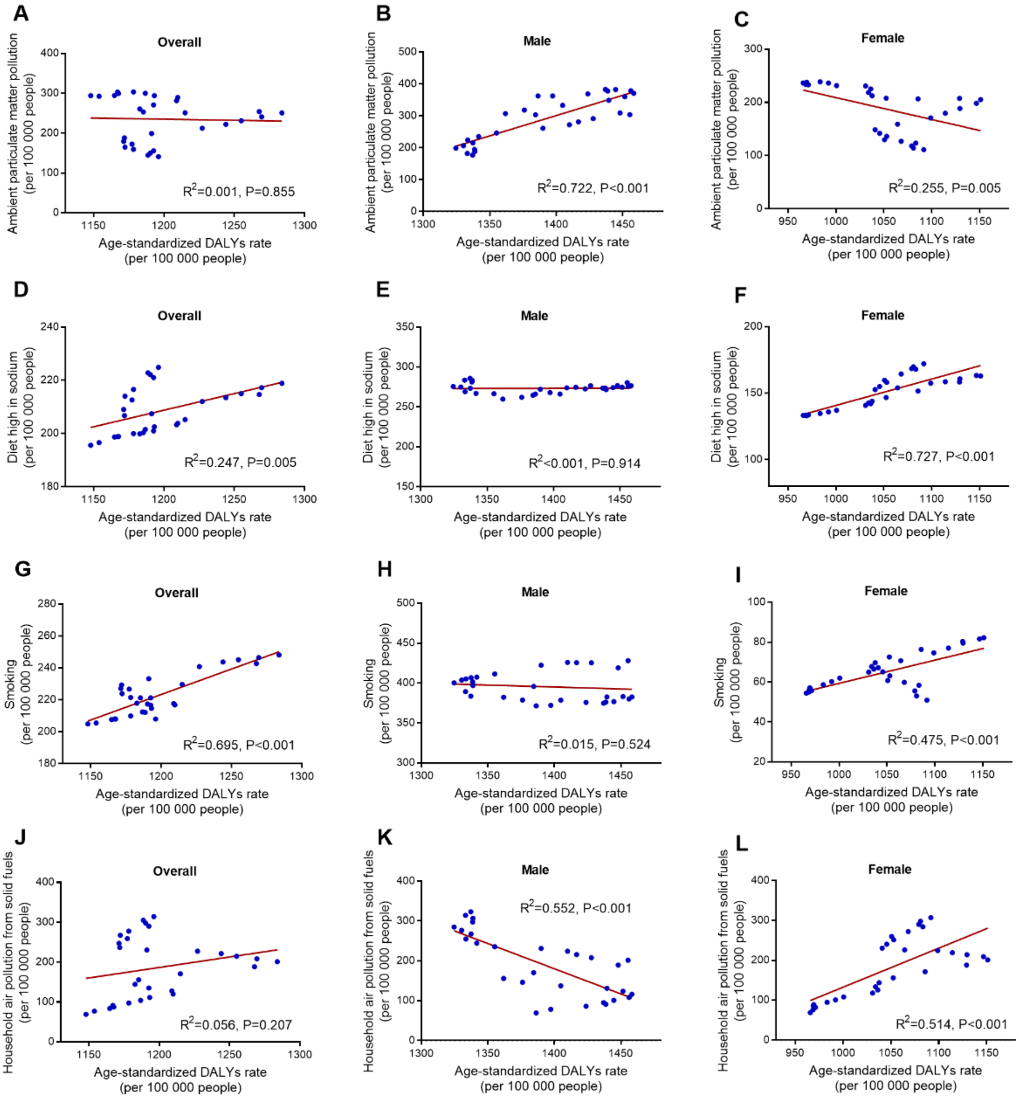

Figure S4. linear regression of risk factors and age-standardized ischemic stroke-related DALYs rate in corresponding years by sex from 1990 to 2019. (A-C) Ambient particulate matter pollution. (D-F) Diet high in sodium. (G-I) Smoking. (J-L) Household air pollution from solid fuels. DALY, disability-adjusted life-year.

Supplementary Figure 5

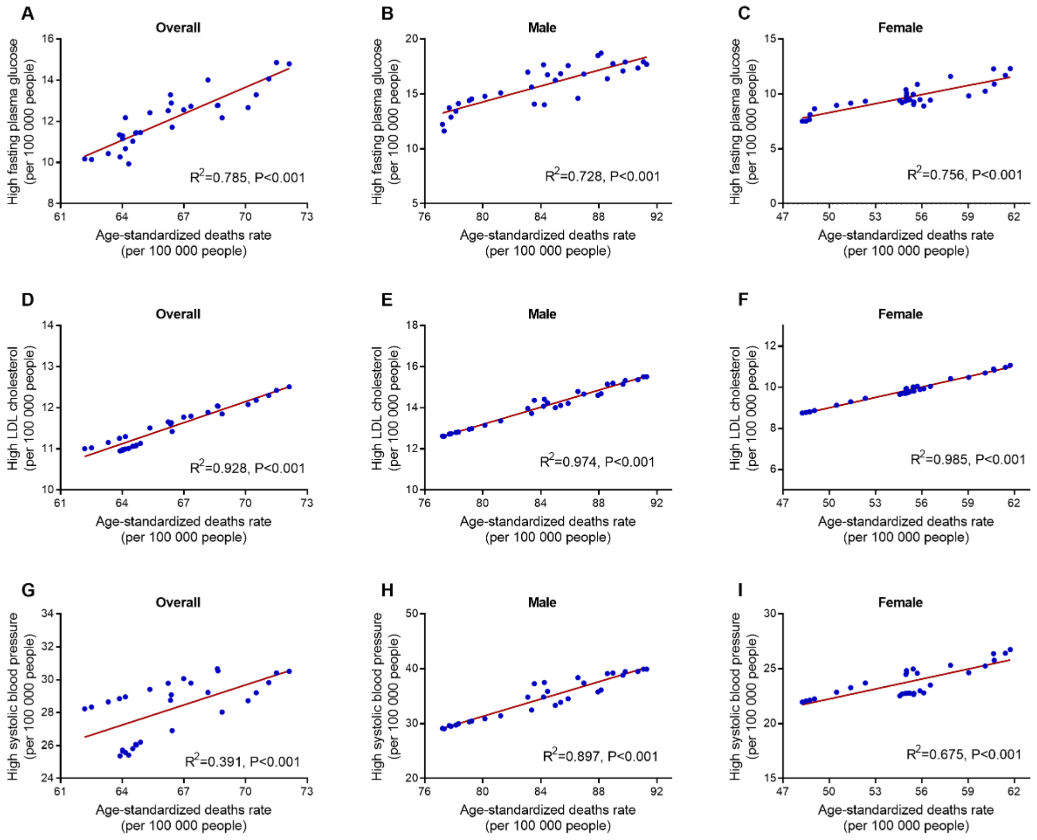

Figure S5. linear regression of risk factors and age-standardized ischemic stroke-related deaths rate in corresponding years by sex from 1990 to 2019. (A-C) High fasting plasma glucose. (D-F) High LDL cholesterol. (G-I) High systolic blood pressure. LDL, low-density lipoprotein.

Supplementary Figure 6

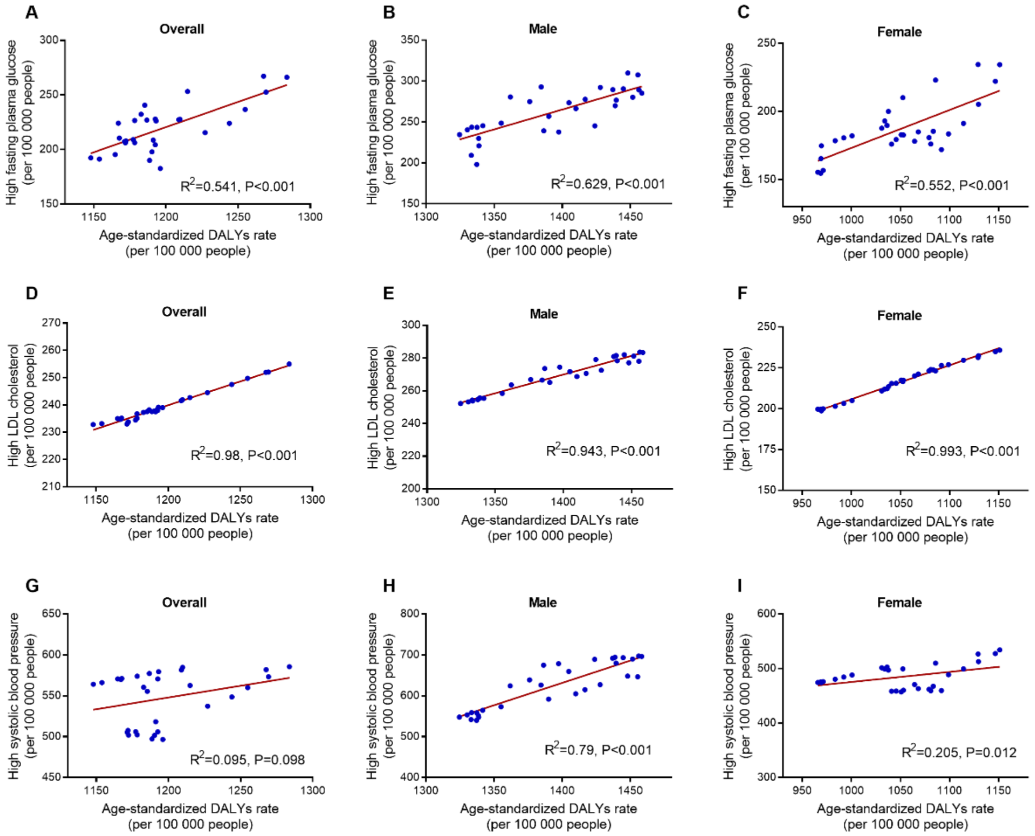

Figure S6. linear regression of risk factors and age-standardized ischemic stroke-related DALYs rate in corresponding years by sex from 1990 to 2019. (A-C) High fasting plasma glucose. (D-F) High LDL cholesterol. (G-I) High systolic blood pressure. DALY, disability-adjusted life-year. LDL, low-density lipoprotein.
